# Cardiopulmonary monitoring in Thai ICUs: results of ICU-RESOURCE I surveys

**DOI:** 10.1186/cc12121

**Published:** 2013-03-19

**Authors:** K Chittawatanarat, A Wattanatham, D Sathaworn, C Permpikul, TSCCM Study Group

**Affiliations:** 1 Chiang Mai University, Chiang Mai, Thailand; 2 Pramongkudklao Hospital, Bangkok, Thailand; 3 Siriraj Hospital, Mahidol University, Bangkok, Thailand; 4 Thai Society of Critical Care Medicine, Bangkok, Thailand

## Introduction

Although rapid progress in ICU monitoring with advanced equipments has been developed, there were limited data on ICU monitoring in Thailand. The objective of this study was to determine the current utilization of monitoring in Thai ICUs.

## Methods

A self-administered questionnaire was developed by the TSCCM research subcommittee. Data verification was processed by an online medical research tools program (OMERET).

## Results

A total of 350 questionnaires were sent to ICUs throughout Thailand. In total, 256 questionnaires were confirmed after being received at the end of June 2012. Of these, 140 filled forms (56.9%) were returned for final analysis. More than 70% of the ICUs had basic hemodynamic monitoring. Less than 10% of general and regional hospitals could perform cardiac output monitoring by thermodilution technique compared with 60% of academic teaching hospitals. New and advanced hemodynamic monitoring techniques such as pulse pressure variation, systolic pressure variation, stroke volume variation, PiCCO, Vigileo-Flo Tract, Pleth variability index device and echocardiography were available only in ICUs of academic teaching hospitals except ultrasound-based techniques including transthoracic and transesophageal echocardiography and USCOM. For respiratory monitoring, all ICUs had a SpO_2 _monitoring device but only one-half of them had end-tidal CO_2 _monitoring. Nearly 80% of ventilator support in participating ICUs was capable of displaying graphic waveform monitoring. Only 43.6% of participating ICUs had a ventilator machine that could calculate lung mechanics data. Advanced respiratory monitoring such as EIT and esophageal pressure monitoring are available only in ICUs of academic teaching hospitals. There was no ICU in Thailand that was capable of measuring extravascular lung water. None of the Thai ICUs used transcutaneous PCO_2_, near-infrared spectroscopy, gut mucosal tonometry and sublingual sidestream darkfield for tissue perfusion monitoring. Only four ICUs had transcutaneous PO_2_. However, measuring the level of lactate as one of the tissue perfusion markers was routinely performed in about 50% of the ICUs.

## Conclusion

There were variations in monitoring performance in Thai ICUs. These vary by type of hospital. Academic ICUs had a tendency for advance monitoring in overall aspects. Some advance monitoring used in developed countries is also unavailable in Thailand.

